# Immune Mediators of protective and pathogenic immune responses in patients with mild and fatal human monocytotropic ehrlichiosis

**DOI:** 10.1186/1471-2172-13-26

**Published:** 2012-05-21

**Authors:** Nahed Ismail, David H Walker, Purnima Ghose, Yi-Wei Tang

**Affiliations:** 1 Department of Pathology, University of Pittsburgh, Pittsburgh, PA, USA; 2 Department of Pathology, University of Texas Medical Branch, Galveston, TX, USA; 3 Department of Pathology, Meharry Medical College, Nashville, TN, USA; 4 Departments of Pathology and Medicine, Memorial Sloan-Kettering Cancer Center, New York, NY, USA; 5 Department of Pathology, University of Pittsburgh, S739 Scaife Hall, 3550 Terrace St, 15261, Pittsburgh, PA, USA

**Keywords:** Human ehrlichiosis, Toxic shock, T cells, Cytokines, Chemokines, Apoptosis, Ehrlichiosis, Pro-inflammatory cytokines, Death Receptors, Th1 response

## Abstract

**Background:**

*Ehrlichia chaffeens*is is a bacterial pathogen that causes fatal human monocytic ehrlichiosis (HME) that mimic toxic shock-like syndrome. Murine studies indicate that over activation of cellular immunity followed by immune suppression plays a central role in mediating tissue injury and organ failure during fatal HME. However, there are no human studies that examine the correlates of resistance or susceptibility to severe and fatal HME.

**Results:**

In this study, we compared the immune responses in two patients with mild/non fatal and severe/fatal HME who had marked lymphopenia, thrombocytopenia and elevated liver enzymes. The levels of different immunological factors in the blood of those patients were examined and compared to healthy controls. Our data showed that fatal HME is associated with defective production of Th1 cytokines such as ( IFNγ and IL-2), increased anti-inflammatory (IL-10 and IL-13) and pro-inflammatory (TNF-α, IL-1α, IL-1β, and IL-6) cytokines, increased levels of macrophages, T cells, and NK cells chemokines such as MCP-1, MIP-1α, MIP-1β, but not RANTES and IP-10, increased levels of neutrophils chemokine and growth factor (IL-8 and G-CSF), and elevated expression of tumor necrosis factor receptor (TNFR), and toll like receptors 2 and 4 compared to patients with non fatal HME and healthy controls.

**Conclusions:**

Fatal *Ehrlichia*-induced toxic shock is associated with defective Th1 responses, possible immune suppression mediated by IL-10. In addition, marked leukopenia observed in patients with fatal disease could be attributed to enhanced apoptosis of leukocytes and/or elevated chemokine production that could promote migration of immune cells to sites of infection causing tissue injury.

## Background

Human monocytic ehrlichiosis is a prevalent tick borne bacterial infection of humans caused by the obligately intracellular bacterium *E. chaffeensis*, a member of the family α-protobacteria [1-3]*.* The disease is associated with significant mortality (~2.7% mortality rate and 20% in hospitalized patients) and is characterized by a rapidly progressive shock-like syndrome preceding death in patients with fatal illness [4,5]. Histopathologic studies in patients and murine models of fatal monocytotropic ehrlichiosis have found widespread extensive necrosis and apoptosis in different organs despite the paucity of infected cells in human peripheral blood [2,6,7]. In immunocompromised patients, similar pathology occurs but in association with an overwhelming infection [8,9]. These pathologic features and the presence of profound shock in fatal cases of HME have led to the hypothesis that fatal disease in immunocompetent individuals is an immune-mediated disease while those in immunocompromised patients could be mediated directly by bacteria inducing tissue damage.

The main target cells for *E. chaffeensis* are monocytes and macrophages; however, other cell types such as endothelial cells and hepatocytes have been observed to be infected in murine ehrlichial infection [3]. Murine studies have shown that effective killing of intracellular ehrlichiae requires IFN-γ, which is usually contributed by natural killer T cells and CD4 + Th1 lymphocytes [7,10-17]. However, the number of lymphocytes, mainly CD4 + T cells, declines dramatically in both humans and animals during fatal disease. Thus, insufficient production of IFN-γ by these cells, and thus insufficient activation of monocytes/macrophages and their intracellular bactericidal mechanisms, are thought to be the reasons for severe course of infection [7,10-13]. Furthermore, in the animal models of fatal ehrlichiosis, development of tissue injury is associated with expansion of NK cells and antigen specific CD8 + T cells as well as their migration to peripheral sites of infection such as liver [12,18]. CD8 + T cells and NK cells not only cause severe tissue injury, but also suppress proliferation of CD4+ Th1 cells and mediate apoptosis of CD4 + T cells, which could account for defective Th1 responses and lymphopenia in fatal ehrlichiosis [12,18].

Although these murine studies reveal immunologic and pathologic dysfunction that could be attributed to soluble mediators, there are no published data in patients with mild/non fatal and severe/fatal disease regarding the potential pathogenic contribution of these soluble mediators in HME. In the present report, we measured the serum levels of cytokines and chemokines as well as the expression of toll like receptors and apoptotic receptors that are involved in differential activation of the immune system and apoptosis of host cells in patients with non fatal or fatal HME. Our data reveal that fatal *Ehrlichia-*induced shock-like syndrome is associated with substantial inflammatory responses and weak protective type-1 immune responses. Furthermore, our data suggest that TLR4 and TNFR may play a role in the development of excessive inflammatory responses and apoptotic cell death in fatal HME. These findings have important implications for the pathogenesis of HME in humans.

## Case reports

### Patient 1

A *7* year old patient with acute lymphocytic leukemia (ALL), who has been in complete remission, was admitted to the hospital after 4 days of persistent fever for which he had been treated as an outpatient with ceftriaxone. Upon admission, his laboratory studies revealed marked leukopenia (WBC count, 500/μl), anemia (Hgb, 11.5 g/dl), thrombocytopenia (platelet count, 164,000/μl), neutropenia (absolute neutrophil count of 420/μl), and increased liver enzymes (ALT 636 U/L and AST 1726 U/L). Blood culture was negative for bacterial and fungal infections. However, *Ehrlichia chaffeensis* DNA was detected in the blood by PCR, but ehrlichiae culture was negative. The acute serum contained no antibodies to *Ehrlichia*. There had been no known tick bite; however, the patient had recently returned from camp in a wooded area known to harbor ticks. He was placed on broad spectrum antibiotics: cefepime, vancomycin, levofloxacin and doxycycline. CT scan of the chest demonstrated right lower lobe pneumonia with moderate right pleural effusion. Cerebrospinal fluid contained a normal concentration of protein and glucose. Shortly after admission, he developed increasing respiratory distress and hypoxia, which necessitated his transfer to the pediatric intensive care unit (PICU). Later, the patient had increasing respiratory distress and began having seizure-like activity, developed significant hyperkalemia, and respiratory and metabolic acidosis. Despite prolonged resuscitation, the patient’s condition deteriorated with multiorgan failure syndrome, and septic shock. He died on day 6 after his admission to the hospital. The cause of death was attributed to septic shock secondary to ehrlichiosis. Autopsy examination showed; 1) cardiomegaly with diffuse focal calcifications in both ventricles; 2) diffuse alveolar damage; 3) hepatic periportal inflammation and focal fibrosis; and 4) lymphocytic depletion in the spleen and lymph nodes. The latter was consistent with presence of leukopenia.

### Patient 2

A 24 year old healthy male presented with a 3 day history of fever, myalgias, and bifrontal headache. Cranial CT was normal. A history of tick exposure during outdoor activities was obtained. CSF culture detected no bacteria or viruses, but contained mild pleocytosis and elevated protein concentration. All laboratory tests including white blood cell count, urinanalysis, and chest radiograph were normal. Serum concentrations of hepatic enzymes were slightly elevated (ALT 55 U/L and AST 63 U/L). The patient was treated with broad spectrum antibiotics and an antiviral drug (vancomycin, ceftriaxone, doxycycline, and acyclovir). *Ehrlichia chaffeensis* DNA was detected in the blood by PCR. The patient was treated with doxycycline for 10 days and remained afebrile with resolution of his frontal headache.

## Methods

### Patients, healthy controls and blood samples

Blood and serum samples were collected from a patient with fatal HME, a patient with mild HME as well as gender- and age-matched healthy volunteers. The serum and blood samples from patient were collected upon hospital admission and thus represent the acute or early phase of illness, a time when ongoing inflammatory responses were likely to be at a maximum. Patient specimens and medical informations that are reported in this study have been collected with the approval of the office of research, Institutional Review Board (IRB) at Meharry Medical College. For patient samples, no consent form is obtained. This study was considered by the IRB as an exempted study, i.e. research that involve the collection of records and diagnostic specimens that are submitted to the clinical laboratory at Vanderbilt Medical Center for diagnostic purposes. Residual samples that remain after performing the required laboratory diagnostic tests are considered as discarded samples and thus used in this study. Patient samples and medical data were collected and recorded in a manner that subjects cannot be identified, directly or through any identifiers linked to the subjects. Blood and sera from healthy controls were collected under the approved IRB protocol and the informed consent was obtained from healthy individuals.

Total mRNA was extracted from peripheral mononuclear cells, and samples were stored at −80°C until processed. Serum and blood samples were collected from healthy individuals as controls.

### Laboratory confirmation of HME

A two-step colorimetric microtiter plate PCR assay capable of detecting and discriminating medically important *Ehrlichia* species was used as described before [19,20]. Isolation of *Ehrlichia* from patients' blood and strain identification were performed as described previously [3-5].

#### Measurement of cytokines and chemokines in sera

Serum levels of 27 cytokines and chemokines including IFN–γ, IL-1α, IL–1β, IL-2, IL-4, IL-5, IL-6, IL-7, IL-10, IL-12, IL-13, and IL-17, the growth factors, G-CSF, and GM-CSF, and chemokines, IL-8, MCP-1, MIP-1α, MIP-1β, and RANTES, were measured in serum samples by multiplex capture ELISA (Luminex System; Luminex), according to the manufacturer's directions. Data were expressed as mean and SD of triplicate measurements of each cytokine or chemokine in the serum samples. Data were measured in Pg/ml.

### Detection of immune response markers in peripheral blood leukocytes by quantitative real time PCR

Total RNA was extracted from human blood using PureLink RNA Mini-extraction Kit (Invitrogen). The quality of RNA was checked before cDNA preparation. Reverse transcription of 1μg of RNA was carried out with the iScript cDNA Synthesis Kit (Invitrogen, Wisc., USA), following the manufacturer’s protocols. The relative expression levels of genes of interest were studied by real-time PCR using My iQ and SYBR green detection system (Bio-Rad TM, USA). The primer sequences are summarized in Table 1. At the end of each reaction, a melting-curve analysis was performed to confirm the absence of primer-dimers. The data were analyzed using the 2 – ΔΔ Ct method with Ct denoting the threshold cycle of PCR amplification at which product is first detected by fluorescence. Expression levels of every gene were normalized to the expression level of GAPDH.

**Table 1:** Primer used for Reverse Transcriptase (RT-PCR)-polymerase chain reaction of cytokines, death receptors, and apoptosis markers in HME patients and controls

**Name**	**Primer sequence**	**Product size (bp)**	**GenBank Accession numbers**
**TLR2**	F: 5'-GGA AGA ATC CTC CAA TCA GGC-3'	100	NM_003264
	R: 5'-CTT CTG TGA GCC CTG AGG GA-3'		
**TLR4**	F: 5'-AGC CAC GCA TTC ACA GGG–3',	100	NM_138554
	R: 5'-CAT GGC TGG GAT CAG AGT CC-3'		
**TLR-9**	F: 5’- GTG ACA GAT CCA AGG TGA AGT-3’	487	NM_017442
	R: 5’- CTT CCT CTA CAA ATG CAT CAC T-3’		
**Fas**	F: 5-TGA AGG ACA TGG CTT AGA AGT G-3’	117	NM_000043
	R: 5-GGT GCA AGG GTC ACA GTG TT-3’		
**FasL**	F: 5- GCA GCC CTT CAA TTA CCC AT-3’	100	NM_000639
	R: 5-CAG AGG TTG GAC AGG GAA GAA-3’		
**TNFR p55**	F: 5'-TGT GTC TCC TGT AGT AAA TG-3'	589	NM_001065
	R: 5'-ACG AAT TCC TTC CAG CGC AA-3'		
**GAPDH**	F: 5-GAA GGT GAA GGT CGG AGT C-3’	225	NM_002046
	R: 5-GAA GAT GGT GAT GGG ATT TC-3’		

## Results

### Cytokine and chemokine profile in the blood

**Figure 1:**
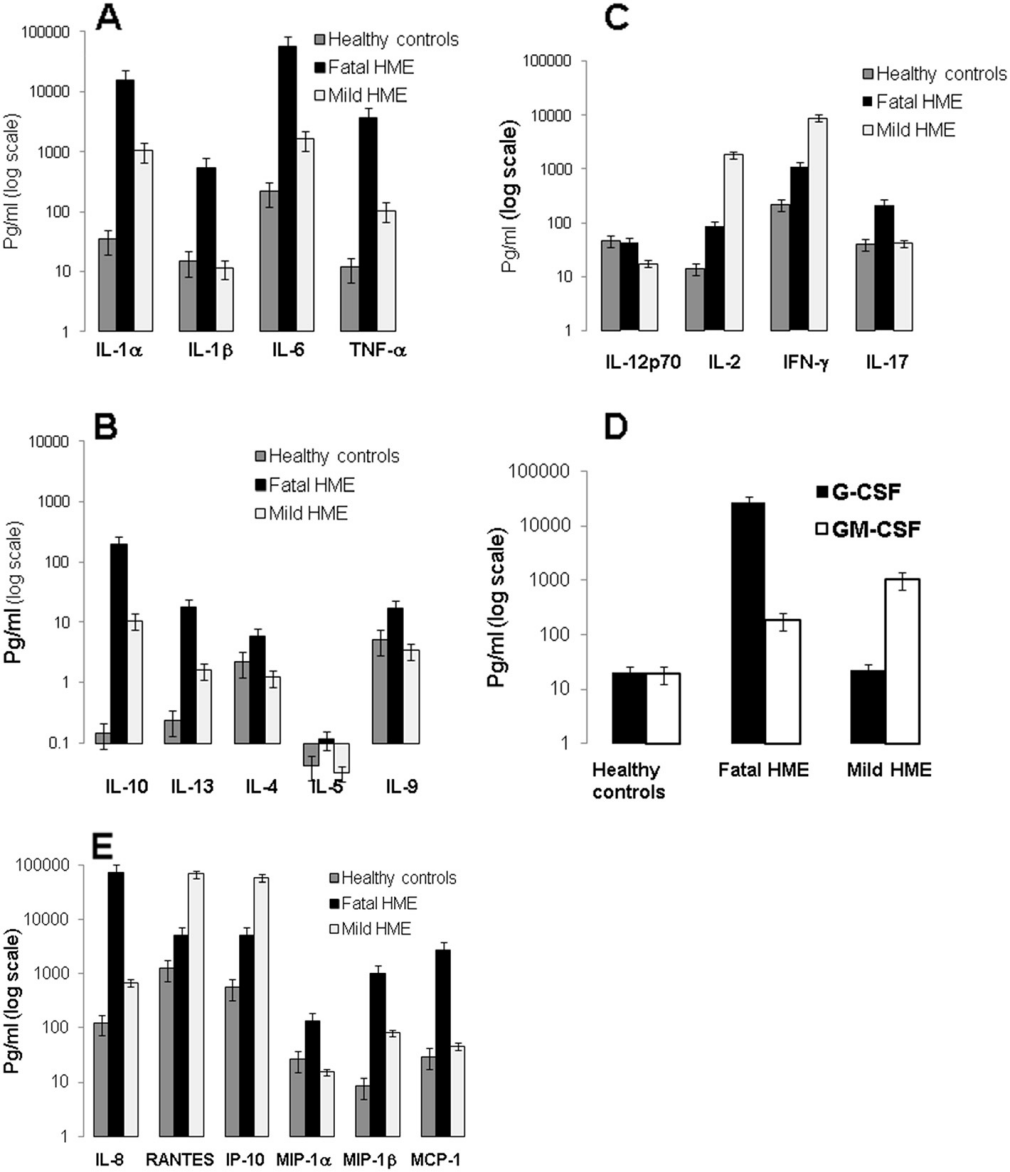
**Levels of cytokines and chemokines in the serum samples from healthy controls (4 pooled samples), patient with severe/fatal or patient with mild/non fatal HME obtained upon hospital admission before antibiotic treatment.** Several cytokines and chemokines described in the Materials and Methods were measured by ELISA and presented in figures **A-E**. Data were expressed as mean and SD of triplicate measurements of each cytokine or chemokine in the serum samples. Data were measured in Pg/ml.

In the serum samples obtained at admission or shortly afterwards, the pro- and anti-inflammatory cytokines and chemokines that we measured fell into five groups including: pro-inflammatory cytokines (IL-1α, IL-1β, IL-6, IL-17, TNF-α), Th1 cytokines (IL-2, IFN-γ, IL-12p70), Th2 cytokines (IL-4, IL-5, IL-9), immunosuppressive or anti-inflammatory cytokines ( IL-10, IL-13), neutrophil chemoattractant and growth factors (IL-8, G-CSF), and T cell, NK cell, and monocyte ckemokines (eotaxin, IP-10, MCP-1, MIP-1α, MIP-1β, RANTES). Our data show that fatal HME is associated with substantially increased levels of pro-inflammatory cytokines such as IL-1α, IL-1β, TNF-α and IL-6 compared to nonfatal HME, and healthy controls (Figure 1A). The levels of anti-inflammatory cytokines such as IL-10 and IL-13 were substantially increased in fatal disease compared to non-fatal HME and controls (Figure 1B). On the other hand, Th1 response marked by the production of Th1 cytokines; IL-2 and IFN-γ was lower in fatal HME compared to non-fatal HME (Figure 1C). Weak Th1 response in the patient with fatal HME was associated with a slight increase in the levels of Th2 cytokines (IL-4 and IL-9) (Figure 1B) and Th17-cytokine (IL-17) (Figure 1C) when compared to patient with non-fatal HME and healthy controls. Interestingly, the levels of IL-8 (Figure 1D) and G-CSF (Figure 1D), a neutrophil chemoattractant and neutrophil growth factor, were greatly increased in fatal HME compared to nonfatal HME and controls, while the level of GM-CSF was slightly lower in fatal HME than that detected in nonfatal HME (Figure 1D).

Macrophage/monocyte and NK cell chemoattractants such as MCP-1α, MIP-1α, and MIP-1β were highest in fatal HME compared to non-fatal HME and healthy controls (Figure 1E). In contrast, the concentrations of the T cell chemokine RANTES and IFN-γ dependent chemokine (IP-10) were lower in fatal HME than in non-fatal HME, but higher than in healthy controls (Figure 1E). The strong clustering of high levels of pro-inflammatory cytokines and monocyte/NK cell chemokines, but the lower levels of Th1 cytokines, the T cell chemokine RANTES, and anti-inflammatory cytokines suggests that fatal disease is associated with excessive pro-inflammatory, but weak Th1, response at least at the time the patients are hospitalized.

### Correlation of a higher expression of Fas with nonfatal HME while a higher expression of TNFR is associated with fatal disease

**Figure 2:**
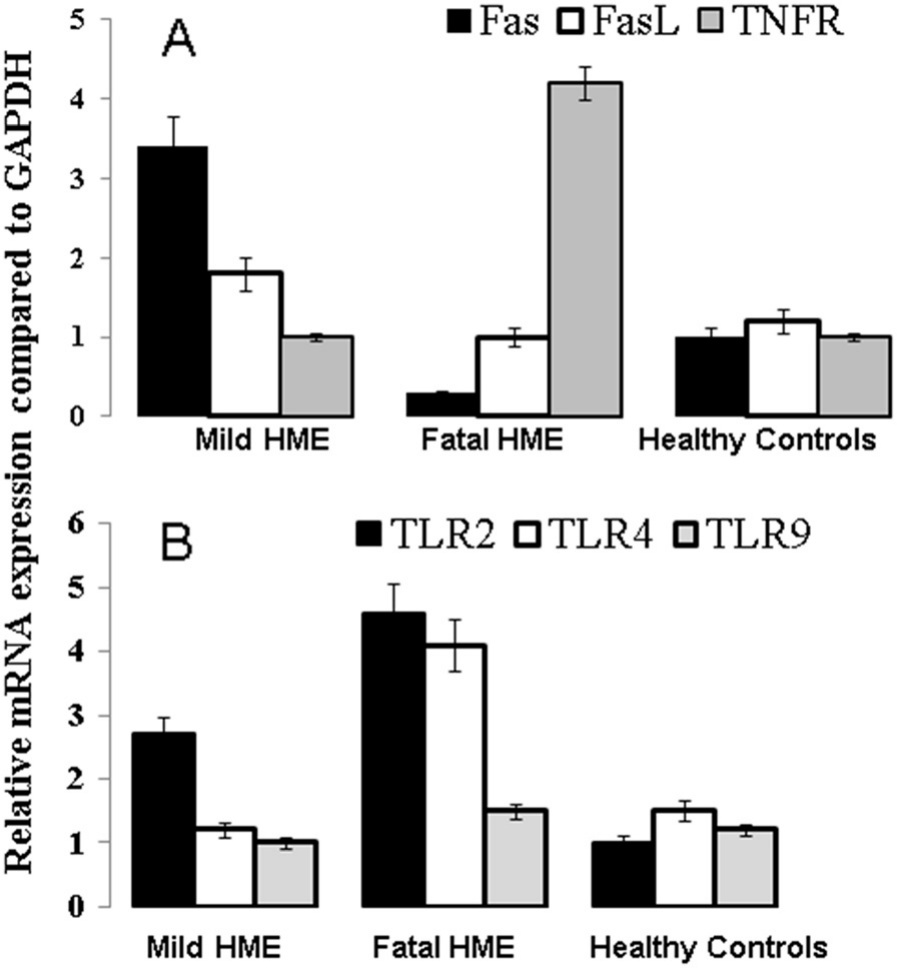
**Expression levels of TLR2, TLR4, TLR9 and death receptors (Fas, FasL, and TNFR) on mononuclear cells from healthy controls and patients with fatal and non fatal HME.** The relative mRNA expression levels of these molecules were normalized to GAPDH as measured by quantitative real time PCR and described in the Materials and Methods. Data were expressed as mean and SD of triplicate measurements of each marker in all patients and healthy control groups.

One of the main clinical manifestations of HME is marked leukopenia, mainly affecting the lymphocyte population. Similarly, in an animal model of fatal ehrlichiosis, death is accompanied by a substantial decrease in the T cell population, mainly CD4 + T cells. As expected, these cases of fatal and nonfatal HME were associated with a decline in the absolute number of lymphocytes, with severe lymphopenia in the fatal case (absolute lymphocyte count: fatal HME = 124/μl and nonfatal HME = 1720/μl). To examine whether the difference in lymphocyte levels in these HME patients was due to apoptosis, we examined the expression of apoptotic markers on peripheral blood leukocytes. Our data show that leukocytes from the patient with milder infection expressed a higher level of FAS mRNA compared to the patient with fatal ehrlichial infection and healthy controls. In contrast, leukocytes from the patient with fatal *Ehrlichia* infection expressed a higher level of TNFR mRNA than that expressed in leukocytes from the patient with milder *Ehrlichia* infection or healthy controls (Figure 2A). There was no difference in mRNA expression of FASL between all groups.

### Increased expression of Toll like receptors-2 and -4 in fatal human Ehrlichiosis

Recognition of specific microbial ligands by toll like receptors (TLRs) has been shown to play a major role in induction of the innate and acquired immune responses against bacteria, viruses, and protozoa [21,22]. We examined whether the above quantitative differences in serum levels of cytokines and chemokines during fatal and nonfatal HME are due to altered signals through TLRs. Our data show that leukocytes from the patient with fatal HME expressed ~ 2-fold higher levels of TLR2 and TLR4 compared to leukocytes from the patient with nonfatal HME or healthy controls (Figure 2B). There was no difference in expression of TLR9 mRNA between all groups (Figure 2B).

## Discussion

Currently, no human study has examined serum cytokine and chemokine profiles in patients with HME who present with either moderate or severe disease. Such studies are critical for enhancing our understanding of the pathophysiology of *Ehrlichia*-induced toxic shock. The development of profound shock, despite the paucity of infected cells in the blood in patients with severe infection with *Ehrlichia*, which lack LPS, has been taken as evidence for subtle immunologic dysfunction attributable to inflammatory mediators. In this study, we compared the levels of circulating pro-inflammatory cytokines and chemokines derived from or involved in the activation of immune cells in acute mild-to-moderate/nonfatal or severe/fatal HME with those seen in uninfected individuals from regions endemic for HME. We could not determine the statistical significance of the differences in the immune responses between the two patient groups due to small sample size. However, we believe that this difference is most likely to be biologically significant based on known levels of these immune molecules in healthy individuals. Our data here showed that fatal HME is associated with dysregulated cytokine and chemokine production with marked elevation of pro-and anti-inflammatory cytokines, and several chemokines that are chemo attractants to NK and T cells. The presence of uncontrolled elevation of inflammatory cytokines in patient with fatal HME could contribute to prolonged activation of *Ehrlichia*-target cells, which could possibly contribute to tissue injury and multi-organ failure.

It is possible that the majority of circulating cytokines and chemokines are produced within peripheral tissues that represent main sites of ehrlichial infection such as liver or lymphoid structures, from where they can leak into the bloodstream. It should be noted that these serum cytokine and chemokine expression levels were studied soon after admission, before progression of the disease or recovery in patients with fatal and nonfatal HME, respectively. Therefore, the immune profile could be considered to be predictive of subsequent adverse events or protection against *Ehrlichia*. In addition, serum cytokine and chemokine expression is measured more easily and is reproducible for rapid clinical diagnostic purposes.

A salient observation in this study was the high levels of IL-8 and G-CSF in patient with acute fatal HME. IL-8, a CXC chemokine, functions as a chemoattractant for neutrophils and T cells and plays an important role in the pathogenesis of septic shock associated with endotoxin [23-26]. G-CSF is shown to be produced as a compensatory mechanism during bacterial infection that are associated with bone marrow suppression [23-26]. G-CSF stimulates the survival, proliferation, differentiation and functions of neutrophils precursors and mature neutrophils [23-26]. However, despite the elevation of IL-8 and G-CSF, we observed low count of neutrophils in the blood of patient with fatal HME. This discordant result could be due to possible migration of neutrophils to the peripheral sites of infection or activation-induced cell death (AICD). In support of the first possibility, patient with fatal HME had a substantial elevation of several chemokines, suggesting that an active migration of inflammatory and immune cells to sites of infection are taking place. Although it is not yet clear whether neutrophils play a protective or pathogenic role during fatal ehrlichial infection in humans, our murine studies showed a correlation between expansion of neutrophils at peripheral sites of infection, ineffective bacterial elimination, and severe pathology in animal model of fatal ehrlichiosis, [27] suggesting that neutrophils may play a pathogenic role in fatal HME.

Activation of monocytes/macrophages killing mechanisms by IFN-γ (Th1 cytokine) is the pivotal step in controlling intracellular ehrlichial infection [7,15,28]. Our data showed that patient with fatal HME had weak Th1 cell-mediated immunity as marked by low serum level of IL-2 and IFN-γ. However, in the absence of kinetic data over the course of illness, it is not possible to determine whether low levels of these Th1 cytokines is due to defective induction of Th1 cells, suppression of T cell proliferation, or AICD. Our murine studies showed that lethal ehrlichial infection induces apoptosis of CD4 + T cells [7,10-13,18]. However, the exact mechanism by which CD4 + T cells undergo apoptosis in this model is not yet clear. TNFR and Fas mediated signals are known to be responsible for apoptosis of host cells during infections [10]. Patient with fatal HME has higher expression of TNFR on the mononuclear cells than that detected in the patient with nonfatal HME, suggesting that signaling via TNFR, but not Fas/FasL signaling, may be relevant to observed weak Th1 response. Interestingly, our murine studies showed that lack of TNFR in lethally infected mice prolonged their survival and abrogated tissue injury [10].

The high levels of serum IL-10 and IL-13 in the patient with fatal HME compared to non fatal HME is consistent with our murine data where IL-10 peaks in the serum at later stages of lethal infection, which occurs before animals succumb to infection [7,10,12]. IL-10 is an immunosuppressive cytokine that inhibits effective elimination of intracellular bacteria and suppresses T cell proliferation [29]. Thus, it is possible that IL-10 overproduction in the patient with fatal HME could be another potential mechanism that account for observed lymphopenia and week Th1 response.

Finally, our data demonstrated a differential TLRs expression during fatal and nonfatal HME with upregulation of TLR2 and TLR4 expression in patient with fatal HME. These data are consistent with TLR expression during sepsis caused by other lipopolysaccharide (LPS) positive Gram negative bacterial infection [21,30]. However, unlike other Gram negative bacteria, E*hrlichia* lack LPS, the natural ligand for TLR4. Therefore, upregulation of TLR4 in fatal disease could be induced by other microbial ligand or by endogenous TLR4 ligand such as the high-mobility group protein (HMGB1), which is secreted upon tissue damage [22].

## Conclusions

In conclusion, defective production of Th1 cytokines (IL-2 and IFN-γ), but increased pro- and anti-inflammatory cytokines and marked lymphopenia in patients with severe and fatal HME is likely to be a key mechanism responsible for progression of ehrlichial infection to toxic shock and multi-organ failure in these patients. This study will not only provide clues for diagnostic markers predictive of disease progression but also a foundation for further studies that analyze the effectiveness of immune-based therapy that target specific inflammatory cytokines or chemokines. The outcome of effective therapeutic approach should involve restoration of the Th1-type cytokine production as well as decrease immune-mediated tissue injury and multi-organ failure in patients with severe and fatal *Ehrlichia* infection.

## Abbreviations

GM-CSF: Granulocyte-macrophage, colony stimulating factor; G-CSF: Granulocyte- colony stimulating factor; HME: Human monocytotropic ehrlichiosis; IFN-γ: interferon gamma; IL: Interleukin; LPS: Lipopolysaccharide; NK: Natural Killer cells; TLR: Toll like receptor; TNF: Tumor necrosis factor; TNFR: Tumor necrosis factor receptors; Th1: T helper cell, type-1.

## Competing Interests

The author(s) declare that they have no competing interests.

## Authors contributions

DWH: have been involved in drafting and revising the manuscript critically for important intellectual content, PG: carried out all the technical experiments performed in this study, YWT: provided the patient serum samples, participated in the design of the study, and involved in revising the manuscript critically, NI: Responsible for the design and coordination of the study, performed data analysis, and wrote the manuscript. NI is also the Corresponding author. All authors read and approved the final manuscript.
